# Synthesis and crystal structure of 2-(2-hy­droxy­phen­yl)-1,3-bis­(4-meth­oxy­benz­yl)-1,3-diazinan-5-ol

**DOI:** 10.1107/S2056989022006508

**Published:** 2022-06-28

**Authors:** Augusto Rivera, Jaime Ríos-Motta, Michael Bolte

**Affiliations:** a Universidad Nacional de Colombia, Sede Bogotá, Facultad de Ciencias, Departamento de Química, Cra 30 No. 45-03, Bogotá, Código Postal 111321, Colombia; b Institut für Anorganische Chemie, J. W. Goethe-Universität Frankfurt, Max-von Laue-Str. 7, 60438 Frankfurt/Main, Germany

**Keywords:** crystal structure, hexa­hydro­pyrimidine, O—H⋯π inter­actions, C—H⋯O inter­actions, intra­molecular hydrogen bond, Mannich-type bases

## Abstract

The redetermined structure of 2-(2-hy­droxy­phen­yl)-1,3-bis­(4-meth­oxy­benz­yl)-1,3-diazinan-5-ol, C_26_H_30_N_2_O_4_, at 173 K has ortho­rhom­bic (*Pbca*) symmetry. It was previously described by Bolte *et al.* [ Private Communication (refcode EWICEV). CCDC, Cambridge, England]. The title compound resulted from the condensation reaction between 1,3-bis­{[(4-meth­oxy­phen­yl)meth­yl]amino}­propan-2-ol and 2-hy­droxy­benzaldehyde in CH_3_OH. The structure exhibits disorder. One of the 4-meth­oxy­benzyl groups, the hy­droxy group bonded to the 1,3-diazinan ring, and the methyl group of the meth­oxy residue are disordered over two orientations, with occupancies of 0.807 (3)/0.193 (3), 0.642 (5)/0.358 (5), and 0.82 (4)/0.18 (4), respectively. The dihedral angles between the mean planes of the central 1,3-diazinan-5-ol and the 4-meth­oxy­phenyl rings (both occupancy components of the disordered ring) are 88.65 (13), 85.79 (14) and 83.4 (7)°. The crystal packing is sustained by C—H⋯O and O—H⋯π inter­actions, giving rise to infinite chains running along the *b-*axis direction.

## Abstract 2

The title compound resulted from the condensation reaction between 1,3-bis­{[(4-meth­oxy­phen­yl)meth­yl]amino}­propan-2-ol and 2-hy­droxy­benzaldehyde in CH_3_OH. The structure exhibits disorder of one of the 4-meth­oxy­benzyl groups, the hy­droxy group bonded to the 1,3-diazinan ring, and the methyl group of the meth­oxy residue. The crystal packing is sustained by C—H⋯O and O—H⋯π inter­actions, giving rise to infinite chains running along the *b-*axis direction.

## 1. Chemical context

Within the framework of a program intended to develop 1,2,3-tris­ubstituted 1,3-diazinan-5-ol derivatives with conformational properties, we were inter­ested in probing the relation between intra­molecular hydrogen bonding and the final conformations of the title compound, which was synthesized by reacting 1,3-bis­{[(4-meth­oxy­phen­yl)meth­yl]amino}­propan-2-ol, easily obtained following the reported method (Rivera, Miranda-Carvajal & Ríos-Motta, 2016 ▸), with 2-hy­droxy­benzaldehyde. Most six-membered heterocycles prefer to adopt chair conformations with equatorially situated substituent groups where the bulky groups attached to the heterocycles generally have a greater preference for the equatorial position than in the case of substituted cyclo­hexane (Wiberg *et al.*, 2018 ▸). Consequently, the ^1^H NMR spectrum (CDCl_3_) of the title compound showed well-resolved signals for the axial and equatorial protons. It is noteworthy that the coupling constants with magnitudes between 2.9 and 3.1 Hz provide a strong evidence of the presence of an axial OH group. In this regard, it has been reported that the presence of an intra­molecular hydrogen bond may stabilize the hydroxyl group in the otherwise non-preferred axial position (Koll *et al.*, 2006 ▸). Therefore, the proton of the OH group in the 5 position of the 1,3-diazinan-5-ol ring might form an intra­molecular hydrogen bond to either one or both endocyclic nitro­gen atoms to stabilize its axial position; however, no such inter­actions were formed. Instead, the crystallographic analysis showed that the intra­molecular hydrogen bonds are observed between the proton of the phenolic OH group and the nitro­gen atoms of the 1,3-diazinan-5-ol ring. 
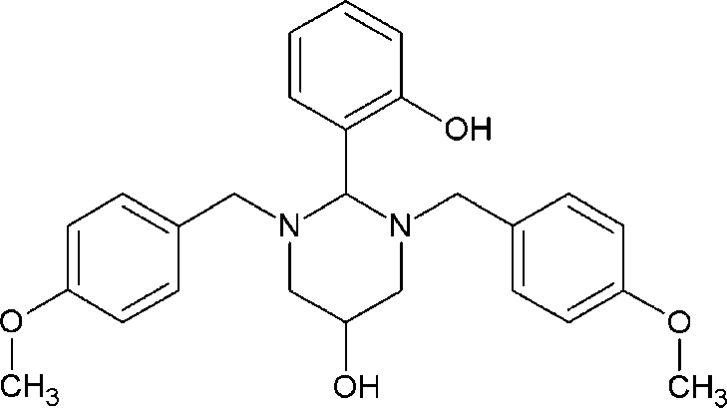


## 2. Structural commentary

**Figure 1:**
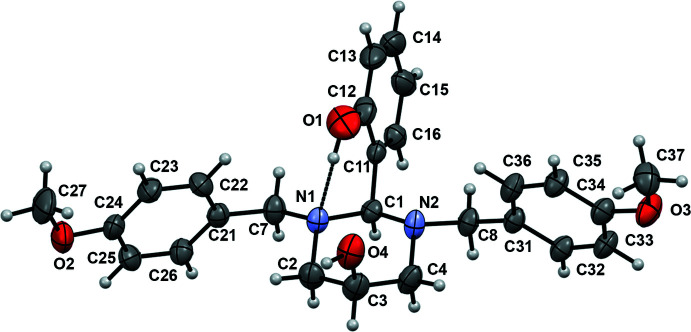
The mol­ecular structure of the title compound. Displacement ellipsoids are drawn at the 50% probability level. The intra­molecular hydrogen bond is shown as a dashed line and, for clarity, only the major disorder components are included.

**Figure 2:**
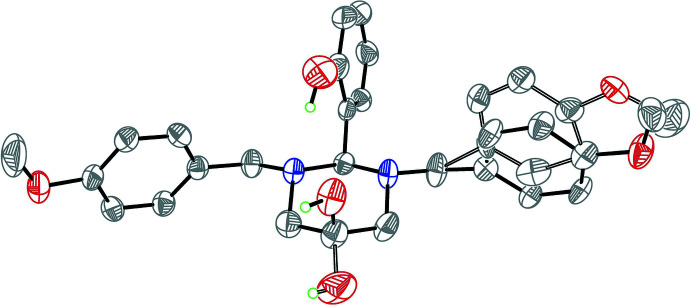
Overlay image of the mol­ecular disorder of the title compound. The major occupancy sites are drawn with full bonds, while the minor occupancy sites with open bonds

The mol­ecular structure of the title compound is shown in Fig. 1 ▸. The 1,3-diazinan-5-ol ring (N1/C1/N2/C4/C3/C2) adopts a chair form with puckering parameters (Cremer & Pople, 1975 ▸) *Q* = 0.562 (3) Å, *θ* = 3.1 (3)°, *φ* = 250 (4)°, *Q*(2) = 0.035 (3) Å and *Q*(3) = −0.561 (3) Å. Atoms C2 and C3 deviate from the mean plane of the other four atoms by −0.242 (3) and 0.249 (3) Å, respectively. Atoms N1 and N2 are essentially tetra­hedral (bond-angle sums are 331.8° for N1 and 330.1° for N2), while the usual Σ_H–N–H_ angle in an ammonia mol­ecule is 321° (Makkos *et al.*, 2021 ▸), with their benzyl substituents in the equatorial positions and axial lone pairs. The dihedral angle between the mean planes of the phenyl rings (C21–C26) and (C31–C36) is 40.41 (19)°. The phenyl ring of the benzyl group bonded to N2 is disordered over two positions (Fig. 2 ▸), with site occupancies refining to 0.807 (3) and 0.193 (3). The aromatic rings of the major (C31–C36) and minor (C31′–C36′) components are roughly parallel, subtending a dihedral angle of 13.5 (7)°. Inter­estingly, the hydroxyl group bonded to the 1,3-diazinan-5-ol ring and the other meth­oxy group are also disordered over two positions with 0.642 (5):0.358 (5) and 0.82 (4):0.18 (4) occupancy ratio, respectively. The C3—O4 and C3—O4′ bond lengths at 1.402 (4) and 1.387 (6) Å are slightly short compared to the normal range (1.421–1.433 Å) for a C_2_—CH—OH group (Allen *et al.*, 1987 ▸).

The dihedral angles between the mean plane of the 1,3-diazinan-5-ol ring [maximum deviation = 0.236 (3) Å] and the C21–C26, C31–C36 and C31′–C36′ phenyl rings of the 1,3- benzyl groups are 88.65 (13), 85.79 (19) and 83.4 (7)°, respectively, whereas the mean plane is rotated by 81.22 (13)° towards the C11–C16 phenyl ring of the 2-hy­droxy­phenyl substituent. The dihedral angles between this phenyl ring and the other two phenyl rings are 55.54 (13)° (C21–C26), 84.27 (19)° (C31–C36) and 77.9 (7)° (C31′–C36′), respectively.

As mentioned above, an intra­mol­ecular O—H⋯N hydrogen bonds is formed between the N1 atom of the 1,3-diazinan-5-ol ring and the OH group of the hy­droxy­phenyl substit­uent, resulting in an *S*(6) graph-set motif (Table1). The N⋯O distance [2.740 (3) Å] is long in comparison with the values observed in related structures [ranging from 2.671 to 2.702 Å; Rivera, Miranda-Carvajal, Ríos-Motta & Bolte, 2016 ▸; Rivera *et al.* 2014 ▸], indicating that the introduction of the hy­droxy­phenyl group in the 2-position of the 1,3-diazinan-5-ol ring decreased the strength of the intra­molecular hydrogen bonds in these compounds.

## 3. Supra­molecular features

In contrast to the supra­molecular structures observed in the previously reported related 1,3-diazinan-5-ol hydrates (Rivera, Miranda-Carvajal, Ríos-Motta & Bolte, 2016 ▸; Rivera *et al.* 2014 ▸), where the water mol­ecules play a significant role in assembling the three-dimensional supra­mol­ecular architecture, the mol­ecular structure of the title compound contains only the unsolvated main mol­ecule.

**Figure 3:**
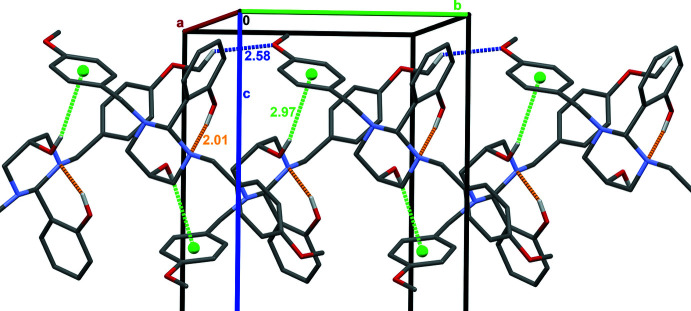
The crystal packing of the title compound, showing the inter­molecular C—H⋯O hydrogen-bonding inter­actions (blue dashed lines) and O—H⋯π (green dashed lines) inter­actions along the *b* axis. Intra­molecular O—H⋯N hydrogen bonds are depicted in orange. Only the H atoms involved in the C—H⋯O, O—H⋯N and O—H⋯π inter­actions are shown for clarity.

In the crystal structure, the mol­ecules are inter­linked *via* C27—H27⋯O3(1 − *x*, 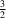 + *y*, 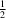 − *z*) non-conventional hydrogen bonds (Fig. 3 ▸, Table 1 ▸) into chains propagating along the *b-*axis direction. Adjacent chains are connected by O—H⋯π inter­actions (Table 1 ▸) [O4—H4⋯*Cg*4 = 2.96 Å O4—H4⋯*Cg*4 = 151°; symmetry code: 1 − *x*, 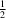 + *y*, 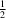 − *z*] involving the major occupancy position of the disordered ring (C31–C36). As a result of the disorder of the aromatic ring, the geometric parameters are less precise than they would be if the ring were not disordered. Although the structure contains three different phenyl rings, only the *Cg*⋯*Cg* distance between two symmetry-related positions (1 − *x*, −*y*, 1 − *z*) of the minor component (C31′–C36′; less than 20% occupancy) of the disordered phenyl ring [3.59 (1) Å] is shorter than 4 Å. It is noteworthy that the inter­planar distance between the symmetry-related main parts of the C31–C36 ring is only 3.66 Å; however, the corresponding *Cg*⋯*Cg* distance is too long at 4.619 (3) Å, indicating a significant horizontal shift of the rings precluding π–π stacking.

## 4. Database survey

A search of the Cambridge Structural Database (CSD, Version 2022.1, last update March 2022; Groom *et al.*, 2016 ▸) for the 1,3-benzyl-1,3-diazinan-5-oxygen unit gave eight hits. Two similar structures have already been deposited with the CSD, namely 1,3-bis­(3-*tert*-butyl-2-hy­droxy-5-methyl­benz­yl)-1,3-diazinan-5-ol monohydrate (ETUYAV; Rivera, Miranda-Carvajal, Ríos-Motta & Bolte, 2016 ▸) and 1,3-bis­(3-*tert*-butyl-2-hy­droxy-5-meth­oxy­benz­yl)hexa­hydro­pyrimidin-5-ol monohydrate (JOGWAF; Rivera *et al.*, 2014 ▸). Inter­estingly, in both of these structures the hydroxyl group bonded to the 1,3-diazinan-5-ol ring is disordered over two positions. The same feature is observed in the title compound. On the other hand, unlike the title compound, ETUYAV and JOGWAF crystallize as hydrates. Unfortunately, because of the multiple disordered parts of the title compound, comparison of the geometric parameters is not reasonable. Other similar compounds with more central ring substituents include 4-allyl-1,3-dibenzyl-1,2,3,4,5,6-hexa­hydro-5-hy­droxy­pyrimidin-2-one (BEMHAC; Enders *et al.*, 1999 ▸) and 5-acet­oxy-1,3,4-tribenzyl-6-(1-(bromo)­phenyl­eth­yl)pyrimidin-2-one (RUTCIS; De Lucca *et al.*, 1997 ▸) and an iron complex of 1,3-bis-(3,5-di-*tert*-butyl-2-hy­droxy­benz­yl)hexa­hydro­pyrimidin-5-ol (MOSYIE; Mendes *et al.*, 2014 ▸) has also been reported.

## 5. Synthesis and crystallization

To a stirred solution of 1,3-bis­{[(4-meth­oxy­phen­yl)meth­yl]amino}­propan-2-ol (661 mg, 2 mmol) in methanol (20 mL) salicyl­aldehyde (0.21 mL, 246 mg, 2 mmol) was added dropwise. The resulting mixture was heated at reflux for 2 h and allowed to cool to room temperature. The solvent was removed under vacuum and the crude solid was washed with cold methanol and dried *in vacuo*. The solid was dissolved in hexa­ne–chloro­form mixture and after standing for several days at room temperature, colorless crystals suitable for X-ray diffraction were obtained. Yield 652 mg (75%), m.p. 413 K.

^1^H NMR (CDCl_3_, 400 MHz) δ 7.15 (*d*, 1H, *J* = 8.60 Hz, Ph-H), 7.12–7.13 (*m*, 1H, Ph-H), 7.11 (*d*, 4H, *J* = 8.80 Hz, Ph-H), 6.99 (*m*, 1H, Ph-H), 6.85 (*d*, 1H, *J* = 7.60 Hz, Ph-H), 6.81 (*d*, 4H, *J* = 8.80 Hz, Ph-H), 3.88 (*s*, 1H, NCHN), 3.76 (*s*, 6H, OCH_3_), 3.72–3.74 (*m*, 1H, CHOH), 3.03 (*d*, 2H, *J* = 12.0 Hz, NCH_2_CHOH), 2.99 (*d*, 2H, *J* = 13.0 Hz, NCH_2_Ph), 2.98 (*d*, 2H, *J* = 13.0 Hz, NCH_2_Ph), 2.22 (*dd*, 2H, *J* = 12.0 and 1.2 Hz, NCH_2_CHOH), 1.60 (*bs*, 1H, OH). The hydrogen atom of a hydroxyl group could not be assigned because of the overlaping and widening of that signal with those due to hydrogen bonds.

Elemental analysis (Thermo Scientific Flash 2000 CHNS/O elemental analyzer): Found, %: C 71.87; H 6.91; N 6.45; O 14.74. C_26_H_30_N_2_O_4_. Calculated, %: C, 71.89; H, 6.91; N, 6.45; O, 14.75.

## 6. Refinement

Crystal data, data collection and structure refinement details are summarized in Table 2 ▸. H atoms bonded to C were refined using a riding model. *U*_iso_ values of methyl H atoms were set to 1.5*U*_eq_(C), while the *U*_iso_ values of H atoms bonded to the remaining C atoms were set to 1.2*U*_eq_(C). The H atom bonded to O in the major occupied site was freely refined. The H atom bonded to O in the minor occupied site was refined using a riding model with *U*_iso_(H) set to 1.5*U*_eq_(O). In addition, the H—O—C—C torsion angle was allowed to refine. The displacement ellipsoids of O4 and O4′ were restrained to be similar. The distances O4—C3 and O4′—C3 were restrained to be similar. Bond lengths and angles in the fragments C24–O2–C27′ and C24–O2–C27 were restrained to be similar. The displacement ellipsoids of O2 and C27/C27′ were restrained to be similar. Bond lengths, angles and displacement parameters in the fragments N2–O3′–C31′–C32′–C33′–C34′–C35′–C36′–C37′–C8′ and N2–O3–C31–C32–C33–C34–C35–C36–C37–C8 were restrained to be similar. The following restraints implemented in *SHELXL* (Sheldrick, 2015) were used to restrain the geometry (SADI, SAME) and *U^ij^* (SIMU, RIGU) of the disordered parts.

## Supplementary Material

Crystal structure: contains datablock(s) I. DOI: 10.1107/S2056989022006508/jq2018sup1.cif

Structure factors: contains datablock(s) I. DOI: 10.1107/S2056989022006508/jq2018Isup2.hkl

Supporting information file. DOI: 10.1107/S2056989022006508/jq2018Isup3.cml

CCDC reference: 2092230

Additional supporting information:  crystallographic information; 3D view; checkCIF report

## Figures and Tables

**Table 1:** Hydrogen-bond geometry (Å, °) *Cg*4 is the centroid of the C31–C36 ring (major occupancy component).

*D*—H⋯*A*	*D*—H	H⋯*A*	*D*⋯*A*	*D*—H⋯*A*
O1—H1⋯N1	0.92 (4)	2.01 (4)	2.740 (3)	135 (3)
C27—H27*B*⋯O3^i^	0.98	2.58	3.342 (10)	134
O4—H4⋯*Cg*4^ii^	0.84	2.96	3.723 (4)	151
O4′—H4′⋯O1^iii^	0.84	2.15	2.898 (6)	148
C27′—H27*D*⋯O4′^iv^	0.98	2.45	3.35 (6)	153
C35′—H35′⋯O2^iii^	0.95	2.55	3.254 (13)	131

Symmetry codes: (i) 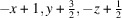 ; (ii) 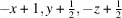 ; (iii) 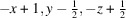 ; (iv) 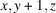 .

**Table 2:** Experimental details

Crystal data	
Chemical formula	C_26_H_30_N_2_O_4_
*M*_r_	434.52
Crystal system, space group	Orthorhombic, *P* *b* *c* *a*
Temperature (K)	173
*a*, *b*, *c* (Å)	19.998 (3), 9.7472 (9), 23.540 (3)
*V* (Å^3^)	4588.5 (10)
*Z*	8
Radiation type	Mo *K*α
μ (mm^−1^)	0.09
Crystal size (mm)	0.22 × 0.19 × 0.16

Data collection	
Diffractometer	Stoe IPDS II two-circle
Absorption correction	Multi-scan (*X-AREA*; Stoe & Cie, 2001 ▸)
*T*_min_, *T*_max_	0.664, 1.000
No. of measured, independent and observed [*I* > 2σ(*I*)] reflections	18262, 4311, 2335
*R*_int_	0.081
(sin θ/λ)_max_ (Å^−1^)	0.609

Refinement	
*R*[*F* ^2^ > 2σ(*F* ^2^)], *wR*(*F* ^2^), *S*	0.060, 0.109, 0.95
No. of reflections	4311
No. of parameters	399
No. of restraints	547
H-atom treatment	H atoms treated by a mixture of independent and constrained refinement
Δρ_max_, Δρ_min_ (e Å^−3^)	0.16, −0.16

Computer programs: *X-AREA* (Stoe & Cie, 2001 ▸), *SHELXS* (Sheldrick, 2008 ▸), *SHELXL2019/2* (Sheldrick, 2015 ▸), and *XP* in *SHELXTL-Plus* (Sheldrick, 2008 ▸).

## supplementary crystallographic information

### Crystal data

**Table:**

C_26_H_30_N_2_O_4_	*D*_x_ = 1.258 Mg m^−^^3^
*M**_r_* = 434.52	Mo *K*α radiation, λ = 0.71073 Å
Orthorhombic, *P**b**c**a*	Cell parameters from 7737 reflections
*a* = 19.998 (3) Å	θ = 3.4–25.6°
*b* = 9.7472 (9) Å	µ = 0.09 mm^−^^1^
*c* = 23.540 (3) Å	*T* = 173 K
*V* = 4588.5 (10) Å^3^	Block, colourless
*Z* = 8	0.22 × 0.19 × 0.16 mm
*F*(000) = 1856	

### Data collection

**Table:**

Stoe IPDS II two-circle diffractometer	2335 reflections with *I* > 2σ(*I*)
Radiation source: Genix 3D IµS microfocus X-ray source	*R*_int_ = 0.081
ω scan	θ_max_ = 25.7°, θ_min_ = 3.4°
Absorption correction: multi-scan (X-Area; Stoe & Cie, 2001)	*h* = −24→22
*T*_min_ = 0.664, *T*_max_ = 1.000	*k* = −11→11
18262 measured reflections	*l* = −24→28
4311 independent reflections	

### Refinement

**Table:**

Refinement on *F*^2^	Primary atom site location: structure-invariant direct methods
Least-squares matrix: full	Secondary atom site location: difference Fourier map
*R*[*F*^2^ > 2σ(*F*^2^)] = 0.060	Hydrogen site location: mixed
*wR*(*F*^2^) = 0.109	H atoms treated by a mixture of independent and constrained refinement
*S* = 0.95	*w* = 1/[σ^2^(*F*_o_^2^) + (0.0335*P*)^2^] where *P* = (*F*_o_^2^ + 2*F*_c_^2^)/3
4311 reflections	(Δ/σ)_max_ < 0.001
399 parameters	Δρ_max_ = 0.16 e Å^−^^3^
547 restraints	Δρ_min_ = −0.16 e Å^−^^3^

### Special details

**Table:**

Geometry. All esds (except the esd in the dihedral angle between two l.s. planes) are estimated using the full covariance matrix. The cell esds are taken into account individually in the estimation of esds in distances, angles and torsion angles; correlations between esds in cell parameters are only used when they are defined by crystal symmetry. An approximate (isotropic) treatment of cell esds is used for estimating esds involving l.s. planes.

### Fractional atomic coordinates and isotropic or equivalent isotropic displacement parameters (Å^2^)

**Table:**

	*x*	*y*	*z*	*U*_iso_*/*U*_eq_	Occ. (<1)
N1	0.64494 (9)	0.3740 (2)	0.25570 (9)	0.0342 (5)	
O1	0.58538 (11)	0.4927 (3)	0.34826 (13)	0.0696 (7)	
H1	0.5815 (18)	0.459 (4)	0.3119 (17)	0.091 (15)*	
O4	0.50732 (14)	0.3007 (3)	0.23995 (15)	0.0555 (13)	0.642 (5)
H4	0.487042	0.337692	0.212717	0.083*	0.642 (5)
O4'	0.5371 (3)	0.1431 (7)	0.1729 (3)	0.081 (3)	0.358 (5)
H4'	0.499929	0.107787	0.180638	0.121*	0.358 (5)
C1	0.66421 (11)	0.2731 (2)	0.30042 (11)	0.0317 (6)	
H1A	0.703034	0.217195	0.286952	0.038*	
C2	0.61862 (13)	0.3081 (3)	0.20410 (13)	0.0457 (7)	
H2A	0.653972	0.251314	0.186348	0.055*	
H2B	0.604863	0.379158	0.176434	0.055*	
C3	0.55947 (13)	0.2191 (3)	0.21893 (13)	0.0464 (7)	
H3	0.543834	0.170613	0.183932	0.056*	0.642 (5)
H3'	0.522283	0.276920	0.234218	0.056*	0.358 (5)
C4	0.58025 (14)	0.1144 (3)	0.26241 (13)	0.0477 (8)	
H4A	0.541151	0.057974	0.273339	0.057*	
H4B	0.614496	0.052832	0.245923	0.057*	
C7	0.70390 (12)	0.4597 (3)	0.24109 (13)	0.0418 (7)	
H7A	0.739601	0.399947	0.225735	0.050*	
H7B	0.721159	0.503354	0.276125	0.050*	
C11	0.68338 (11)	0.3498 (2)	0.35392 (11)	0.0330 (6)	
C12	0.64143 (14)	0.4514 (3)	0.37613 (13)	0.0475 (8)	
C13	0.65800 (17)	0.5142 (3)	0.42826 (15)	0.0586 (9)	
H13	0.629526	0.582640	0.443749	0.070*	
C14	0.71505 (17)	0.4768 (3)	0.45672 (15)	0.0584 (9)	
H14	0.725369	0.519152	0.491998	0.070*	
C15	0.75702 (15)	0.3802 (3)	0.43525 (13)	0.0495 (8)	
H15	0.796674	0.355656	0.455011	0.059*	
C16	0.74101 (12)	0.3179 (3)	0.38395 (12)	0.0378 (6)	
H16	0.770627	0.250933	0.368871	0.045*	
C21	0.68809 (11)	0.5699 (3)	0.19816 (11)	0.0350 (6)	
C22	0.63905 (13)	0.6682 (3)	0.20784 (12)	0.0419 (7)	
H22	0.615087	0.666722	0.242676	0.050*	
C23	0.62426 (13)	0.7679 (3)	0.16811 (13)	0.0433 (7)	
H23	0.590397	0.833729	0.175748	0.052*	
C24	0.65872 (13)	0.7720 (3)	0.11727 (12)	0.0403 (7)	
O2	0.64754 (11)	0.8643 (2)	0.07398 (9)	0.0589 (6)	
C27	0.5966 (7)	0.9663 (14)	0.0835 (5)	0.072 (3)	0.82 (4)
H27A	0.553374	0.921016	0.089000	0.108*	0.82 (4)
H27B	0.594104	1.027474	0.050510	0.108*	0.82 (4)
H27C	0.607802	1.019893	0.117417	0.108*	0.82 (4)
C27'	0.5806 (14)	0.923 (6)	0.070 (2)	0.065 (8)	0.18 (4)
H27D	0.567101	0.958041	0.107442	0.097*	0.18 (4)
H27E	0.548981	0.851932	0.057900	0.097*	0.18 (4)
H27F	0.580585	0.998125	0.042519	0.097*	0.18 (4)
C25	0.70868 (13)	0.6764 (3)	0.10696 (13)	0.0439 (7)	
H25	0.733259	0.679525	0.072447	0.053*	
C26	0.72262 (12)	0.5768 (3)	0.14690 (12)	0.0395 (7)	
H26	0.756625	0.511338	0.139212	0.047*	
N2	0.60744 (9)	0.1826 (2)	0.31273 (10)	0.0383 (6)	
O3	0.41646 (11)	−0.2103 (2)	0.46241 (12)	0.0543 (8)	0.807 (3)
C31	0.56972 (15)	−0.0008 (3)	0.37903 (16)	0.0347 (8)	0.807 (3)
C32	0.56991 (16)	−0.1417 (3)	0.38677 (16)	0.0373 (8)	0.807 (3)
H32	0.606369	−0.193688	0.372450	0.045*	0.807 (3)
C33	0.51887 (15)	−0.2086 (3)	0.41458 (15)	0.0410 (9)	0.807 (3)
H33	0.520469	−0.305216	0.419593	0.049*	0.807 (3)
C34	0.4650 (3)	−0.1337 (7)	0.4353 (4)	0.0414 (12)	0.807 (3)
C35	0.46267 (16)	0.0054 (4)	0.42766 (18)	0.0427 (9)	0.807 (3)
H35	0.425728	0.056923	0.441423	0.051*	0.807 (3)
C36	0.51522 (17)	0.0710 (4)	0.39942 (16)	0.0433 (9)	0.807 (3)
H36	0.513401	0.167498	0.394115	0.052*	0.807 (3)
C37	0.3584 (3)	−0.1389 (5)	0.4817 (3)	0.0555 (14)	0.807 (3)
H37A	0.327839	−0.203731	0.500109	0.083*	0.807 (3)
H37B	0.371569	−0.067890	0.508933	0.083*	0.807 (3)
H37C	0.335876	−0.096122	0.449219	0.083*	0.807 (3)
C8	0.6279 (2)	0.0714 (4)	0.35223 (17)	0.0386 (10)	0.807 (3)
H8A	0.656337	0.110931	0.382523	0.046*	0.807 (3)
H8B	0.655174	0.003572	0.331143	0.046*	0.807 (3)
O3'	0.3808 (5)	−0.0422 (11)	0.4830 (5)	0.051 (2)	0.193 (3)
C31'	0.5554 (8)	0.0528 (13)	0.3947 (7)	0.038 (2)	0.193 (3)
C32'	0.5373 (7)	−0.0836 (13)	0.3961 (7)	0.0423 (18)	0.193 (3)
H32'	0.562669	−0.150686	0.376320	0.051*	0.193 (3)
C33'	0.4778 (15)	−0.123 (3)	0.4290 (18)	0.046 (2)	0.193 (3)
H33'	0.465784	−0.216326	0.433355	0.055*	0.193 (3)
C34'	0.4398 (7)	−0.0205 (13)	0.4532 (7)	0.045 (2)	0.193 (3)
C35'	0.4582 (6)	0.1174 (12)	0.4500 (6)	0.041 (2)	0.193 (3)
H35'	0.431650	0.186024	0.467596	0.049*	0.193 (3)
C36'	0.5156 (5)	0.1526 (13)	0.4208 (6)	0.039 (2)	0.193 (3)
H36'	0.528306	0.246367	0.418490	0.047*	0.193 (3)
C37'	0.3661 (13)	−0.1853 (19)	0.4926 (14)	0.060 (5)	0.193 (3)
H37D	0.324024	−0.193397	0.513728	0.089*	0.193 (3)
H37E	0.361821	−0.232464	0.456030	0.089*	0.193 (3)
H37F	0.402342	−0.227054	0.514611	0.089*	0.193 (3)
C8'	0.6183 (8)	0.1040 (18)	0.3664 (6)	0.039 (3)	0.193 (3)
H8C	0.642506	0.163498	0.393605	0.047*	0.193 (3)
H8D	0.647309	0.024347	0.358023	0.047*	0.193 (3)

### Atomic displacement parameters (Å^2^)

**Table:**

	*U*^11^	*U*^22^	*U*^33^	*U*^12^	*U*^13^	*U*^23^
N1	0.0291 (10)	0.0343 (11)	0.0394 (14)	−0.0033 (9)	−0.0047 (10)	0.0072 (11)
O1	0.0639 (14)	0.0682 (15)	0.077 (2)	0.0357 (12)	0.0098 (14)	0.0027 (15)
O4	0.0386 (18)	0.053 (2)	0.075 (3)	0.0011 (15)	−0.0129 (17)	0.0146 (18)
O4'	0.080 (5)	0.077 (5)	0.085 (6)	−0.021 (4)	−0.035 (4)	−0.014 (4)
C1	0.0294 (12)	0.0279 (13)	0.0378 (17)	0.0049 (11)	−0.0013 (11)	0.0018 (12)
C2	0.0453 (16)	0.0478 (18)	0.0440 (19)	−0.0053 (13)	−0.0089 (13)	0.0045 (15)
C3	0.0449 (16)	0.0424 (16)	0.052 (2)	−0.0054 (14)	−0.0148 (14)	0.0031 (15)
C4	0.0491 (16)	0.0340 (15)	0.060 (2)	−0.0079 (13)	−0.0141 (15)	0.0048 (15)
C7	0.0340 (13)	0.0427 (17)	0.0488 (19)	−0.0053 (12)	−0.0003 (12)	0.0103 (14)
C11	0.0372 (13)	0.0249 (13)	0.0370 (16)	−0.0019 (11)	0.0059 (12)	0.0048 (12)
C12	0.0516 (17)	0.0371 (16)	0.054 (2)	0.0098 (13)	0.0098 (15)	0.0084 (15)
C13	0.082 (2)	0.0371 (17)	0.057 (2)	0.0042 (17)	0.0285 (19)	−0.0050 (16)
C14	0.080 (2)	0.048 (2)	0.047 (2)	−0.0206 (18)	0.0063 (19)	−0.0047 (17)
C15	0.0560 (17)	0.0540 (18)	0.0387 (18)	−0.0203 (16)	0.0016 (15)	0.0011 (16)
C16	0.0343 (14)	0.0401 (15)	0.0390 (17)	−0.0060 (12)	0.0031 (12)	0.0030 (14)
C21	0.0317 (13)	0.0351 (14)	0.0383 (17)	−0.0052 (12)	−0.0003 (12)	0.0018 (13)
C22	0.0464 (15)	0.0445 (16)	0.0348 (17)	−0.0016 (13)	0.0090 (13)	0.0006 (14)
C23	0.0508 (17)	0.0333 (15)	0.0457 (19)	0.0063 (13)	0.0039 (14)	0.0010 (14)
C24	0.0474 (16)	0.0311 (14)	0.0425 (19)	−0.0078 (13)	0.0014 (14)	0.0049 (14)
O2	0.0808 (14)	0.0443 (12)	0.0514 (15)	0.0060 (11)	0.0072 (11)	0.0169 (11)
C27	0.098 (5)	0.041 (4)	0.076 (5)	0.015 (4)	0.000 (3)	0.023 (3)
C27'	0.095 (9)	0.034 (15)	0.065 (19)	0.006 (9)	0.001 (9)	0.019 (14)
C25	0.0477 (16)	0.0453 (17)	0.0389 (18)	−0.0069 (14)	0.0129 (13)	0.0034 (14)
C26	0.0375 (14)	0.0373 (15)	0.0438 (19)	0.0013 (12)	0.0046 (12)	0.0047 (14)
N2	0.0321 (11)	0.0356 (12)	0.0473 (15)	−0.0055 (10)	−0.0070 (10)	0.0145 (10)
O3	0.0421 (13)	0.0374 (13)	0.084 (2)	−0.0022 (11)	0.0177 (13)	0.0050 (13)
C31	0.0315 (16)	0.0299 (17)	0.043 (2)	0.0002 (14)	−0.0070 (14)	0.0052 (16)
C32	0.0328 (16)	0.0299 (16)	0.049 (2)	0.0019 (14)	−0.0058 (15)	0.0022 (16)
C33	0.0390 (16)	0.0256 (16)	0.059 (2)	−0.0024 (14)	−0.0003 (15)	0.0016 (16)
C34	0.036 (2)	0.036 (2)	0.052 (3)	−0.0054 (18)	0.000 (2)	0.0018 (18)
C35	0.0372 (17)	0.0326 (17)	0.058 (2)	0.0028 (14)	0.0021 (16)	0.0016 (16)
C36	0.0425 (18)	0.0286 (18)	0.059 (2)	−0.0006 (16)	−0.0040 (17)	0.0074 (17)
C37	0.049 (2)	0.055 (3)	0.063 (3)	0.008 (2)	0.016 (2)	0.011 (3)
C8	0.0358 (18)	0.031 (2)	0.049 (2)	0.0034 (15)	−0.0056 (16)	0.0100 (18)
O3'	0.053 (4)	0.044 (4)	0.057 (5)	−0.006 (4)	0.006 (4)	0.000 (4)
C31'	0.034 (3)	0.030 (3)	0.049 (4)	0.000 (3)	−0.010 (3)	0.009 (3)
C32'	0.041 (3)	0.032 (3)	0.054 (4)	−0.002 (3)	−0.002 (3)	0.004 (3)
C33'	0.043 (4)	0.035 (4)	0.060 (4)	−0.007 (3)	0.003 (4)	0.003 (4)
C34'	0.043 (4)	0.036 (4)	0.056 (4)	−0.004 (3)	−0.002 (3)	0.002 (3)
C35'	0.039 (4)	0.033 (4)	0.051 (5)	−0.002 (3)	−0.011 (4)	0.003 (4)
C36'	0.036 (4)	0.031 (4)	0.050 (5)	0.000 (4)	−0.013 (4)	0.006 (4)
C37'	0.056 (9)	0.048 (7)	0.075 (10)	−0.008 (6)	−0.001 (8)	0.004 (6)
C8'	0.034 (4)	0.031 (5)	0.051 (4)	0.001 (4)	−0.008 (4)	0.011 (4)

### Geometric parameters (Å, º)

**Table:**

N1—C2	1.472 (3)	C27—H27C	0.9800
N1—C7	1.486 (3)	C27'—H27D	0.9800
N1—C1	1.491 (3)	C27'—H27E	0.9800
O1—C12	1.360 (4)	C27'—H27F	0.9800
O1—H1	0.92 (4)	C25—C26	1.380 (4)
O4—C3	1.402 (4)	C25—H25	0.9500
O4—H4	0.8400	C26—H26	0.9500
O4'—C3	1.387 (6)	N2—C8	1.486 (4)
O4'—H4'	0.8400	N2—C8'	1.494 (8)
C1—N2	1.467 (3)	O3—C34	1.382 (5)
C1—C11	1.514 (4)	O3—C37	1.428 (5)
C1—H1A	1.0000	C31—C36	1.381 (5)
C2—C3	1.508 (4)	C31—C32	1.385 (4)
C2—H2A	0.9900	C31—C8	1.499 (5)
C2—H2B	0.9900	C32—C33	1.377 (4)
C3—C4	1.504 (4)	C32—H32	0.9500
C3—H3	1.0000	C33—C34	1.389 (7)
C3—H3'	1.0000	C33—H33	0.9500
C4—N2	1.463 (4)	C34—C35	1.369 (7)
C4—H4A	0.9900	C35—C36	1.398 (5)
C4—H4B	0.9900	C35—H35	0.9500
C7—C21	1.508 (4)	C36—H36	0.9500
C7—H7A	0.9900	C37—H37A	0.9800
C7—H7B	0.9900	C37—H37B	0.9800
C11—C16	1.387 (3)	C37—H37C	0.9800
C11—C12	1.400 (4)	C8—H8A	0.9900
C12—C13	1.411 (4)	C8—H8B	0.9900
C13—C14	1.372 (4)	O3'—C34'	1.388 (14)
C13—H13	0.9500	O3'—C37'	1.443 (15)
C14—C15	1.359 (4)	C31'—C32'	1.378 (12)
C14—H14	0.9500	C31'—C36'	1.400 (12)
C15—C16	1.389 (4)	C31'—C8'	1.508 (15)
C15—H15	0.9500	C32'—C33'	1.47 (3)
C16—H16	0.9500	C32'—H32'	0.9500
C21—C22	1.390 (4)	C33'—C34'	1.38 (4)
C21—C26	1.392 (4)	C33'—H33'	0.9500
C22—C23	1.381 (4)	C34'—C35'	1.396 (12)
C22—H22	0.9500	C35'—C36'	1.380 (11)
C23—C24	1.382 (4)	C35'—H35'	0.9500
C23—H23	0.9500	C36'—H36'	0.9500
C24—O2	1.378 (3)	C37'—H37D	0.9800
C24—C25	1.387 (4)	C37'—H37E	0.9800
O2—C27	1.440 (5)	C37'—H37F	0.9800
O2—C27'	1.459 (16)	C8'—H8C	0.9900
C27—H27A	0.9800	C8'—H8D	0.9900
C27—H27B	0.9800		
			
C2—N1—C7	109.8 (2)	O2—C27'—H27D	109.5
C2—N1—C1	112.8 (2)	O2—C27'—H27E	109.5
C7—N1—C1	109.24 (18)	H27D—C27'—H27E	109.5
C12—O1—H1	114 (2)	O2—C27'—H27F	109.5
C3—O4—H4	109.5	H27D—C27'—H27F	109.5
C3—O4'—H4'	109.5	H27E—C27'—H27F	109.5
N2—C1—N1	109.64 (18)	C26—C25—C24	119.9 (3)
N2—C1—C11	109.2 (2)	C26—C25—H25	120.0
N1—C1—C11	109.1 (2)	C24—C25—H25	120.0
N2—C1—H1A	109.6	C25—C26—C21	121.6 (2)
N1—C1—H1A	109.6	C25—C26—H26	119.2
C11—C1—H1A	109.6	C21—C26—H26	119.2
N1—C2—C3	109.9 (2)	C4—N2—C1	113.6 (2)
N1—C2—H2A	109.7	C4—N2—C8	106.1 (2)
C3—C2—H2A	109.7	C1—N2—C8	110.4 (2)
N1—C2—H2B	109.7	C4—N2—C8'	120.4 (8)
C3—C2—H2B	109.7	C1—N2—C8'	111.3 (7)
H2A—C2—H2B	108.2	C34—O3—C37	117.1 (4)
O4'—C3—C4	105.0 (4)	C36—C31—C32	117.3 (3)
O4—C3—C4	110.5 (3)	C36—C31—C8	121.4 (3)
O4'—C3—C2	112.2 (4)	C32—C31—C8	121.2 (3)
O4—C3—C2	109.8 (3)	C33—C32—C31	122.0 (3)
C4—C3—C2	109.3 (2)	C33—C32—H32	119.0
O4—C3—H3	109.0	C31—C32—H32	119.0
C4—C3—H3	109.0	C32—C33—C34	119.5 (4)
C2—C3—H3	109.0	C32—C33—H33	120.2
O4'—C3—H3'	110.1	C34—C33—H33	120.2
C4—C3—H3'	110.1	C35—C34—O3	124.9 (5)
C2—C3—H3'	110.1	C35—C34—C33	120.1 (4)
N2—C4—C3	110.2 (2)	O3—C34—C33	115.0 (5)
N2—C4—H4A	109.6	C34—C35—C36	119.3 (4)
C3—C4—H4A	109.6	C34—C35—H35	120.4
N2—C4—H4B	109.6	C36—C35—H35	120.4
C3—C4—H4B	109.6	C31—C36—C35	121.8 (3)
H4A—C4—H4B	108.1	C31—C36—H36	119.1
N1—C7—C21	112.9 (2)	C35—C36—H36	119.1
N1—C7—H7A	109.0	O3—C37—H37A	109.5
C21—C7—H7A	109.0	O3—C37—H37B	109.5
N1—C7—H7B	109.0	H37A—C37—H37B	109.5
C21—C7—H7B	109.0	O3—C37—H37C	109.5
H7A—C7—H7B	107.8	H37A—C37—H37C	109.5
C16—C11—C12	117.8 (3)	H37B—C37—H37C	109.5
C16—C11—C1	121.6 (2)	N2—C8—C31	113.1 (3)
C12—C11—C1	120.6 (2)	N2—C8—H8A	109.0
O1—C12—C11	121.6 (3)	C31—C8—H8A	109.0
O1—C12—C13	119.0 (3)	N2—C8—H8B	109.0
C11—C12—C13	119.4 (3)	C31—C8—H8B	109.0
C14—C13—C12	120.3 (3)	H8A—C8—H8B	107.8
C14—C13—H13	119.8	C34'—O3'—C37'	113.6 (14)
C12—C13—H13	119.8	C32'—C31'—C36'	120.7 (12)
C15—C14—C13	121.1 (3)	C32'—C31'—C8'	123.3 (12)
C15—C14—H14	119.5	C36'—C31'—C8'	116.0 (11)
C13—C14—H14	119.5	C31'—C32'—C33'	118.4 (17)
C14—C15—C16	119.0 (3)	C31'—C32'—H32'	120.8
C14—C15—H15	120.5	C33'—C32'—H32'	120.8
C16—C15—H15	120.5	C34'—C33'—C32'	119 (2)
C11—C16—C15	122.4 (3)	C34'—C33'—H33'	120.7
C11—C16—H16	118.8	C32'—C33'—H33'	120.7
C15—C16—H16	118.8	C33'—C34'—O3'	124.6 (16)
C22—C21—C26	117.3 (2)	C33'—C34'—C35'	121.9 (17)
C22—C21—C7	121.9 (2)	O3'—C34'—C35'	113.5 (11)
C26—C21—C7	120.8 (2)	C36'—C35'—C34'	119.1 (12)
C23—C22—C21	121.7 (3)	C36'—C35'—H35'	120.4
C23—C22—H22	119.1	C34'—C35'—H35'	120.4
C21—C22—H22	119.1	C35'—C36'—C31'	121.2 (11)
C22—C23—C24	120.0 (2)	C35'—C36'—H36'	119.4
C22—C23—H23	120.0	C31'—C36'—H36'	119.4
C24—C23—H23	120.0	O3'—C37'—H37D	109.5
O2—C24—C23	125.3 (2)	O3'—C37'—H37E	109.5
O2—C24—C25	115.2 (3)	H37D—C37'—H37E	109.5
C23—C24—C25	119.4 (3)	O3'—C37'—H37F	109.5
C24—O2—C27	116.8 (4)	H37D—C37'—H37F	109.5
C24—O2—C27'	116.8 (16)	H37E—C37'—H37F	109.5
O2—C27—H27A	109.5	N2—C8'—C31'	115.0 (11)
O2—C27—H27B	109.5	N2—C8'—H8C	108.5
H27A—C27—H27B	109.5	C31'—C8'—H8C	108.5
O2—C27—H27C	109.5	N2—C8'—H8D	108.5
H27A—C27—H27C	109.5	C31'—C8'—H8D	108.5
H27B—C27—H27C	109.5	H8C—C8'—H8D	107.5
			
C2—N1—C1—N2	−54.3 (3)	C22—C21—C26—C25	−0.4 (4)
C7—N1—C1—N2	−176.7 (2)	C7—C21—C26—C25	179.7 (2)
C2—N1—C1—C11	−173.8 (2)	C3—C4—N2—C1	−57.1 (3)
C7—N1—C1—C11	63.8 (3)	C3—C4—N2—C8	−178.6 (2)
C7—N1—C2—C3	179.1 (2)	C3—C4—N2—C8'	167.1 (7)
C1—N1—C2—C3	57.0 (3)	N1—C1—N2—C4	54.3 (3)
N1—C2—C3—O4'	−173.5 (4)	C11—C1—N2—C4	173.8 (2)
N1—C2—C3—O4	63.9 (3)	N1—C1—N2—C8	173.5 (2)
N1—C2—C3—C4	−57.5 (3)	C11—C1—N2—C8	−67.1 (3)
O4'—C3—C4—N2	177.8 (4)	N1—C1—N2—C8'	−165.8 (8)
O4—C3—C4—N2	−63.8 (3)	C11—C1—N2—C8'	−46.4 (8)
C2—C3—C4—N2	57.2 (3)	C36—C31—C32—C33	−1.3 (5)
C2—N1—C7—C21	58.8 (3)	C8—C31—C32—C33	175.7 (3)
C1—N1—C7—C21	−177.1 (2)	C31—C32—C33—C34	0.6 (7)
N2—C1—C11—C16	107.0 (3)	C37—O3—C34—C35	2.8 (10)
N1—C1—C11—C16	−133.2 (2)	C37—O3—C34—C33	−176.7 (5)
N2—C1—C11—C12	−69.9 (3)	C32—C33—C34—C35	0.4 (9)
N1—C1—C11—C12	49.9 (3)	C32—C33—C34—O3	179.9 (5)
C16—C11—C12—O1	177.4 (3)	O3—C34—C35—C36	179.9 (6)
C1—C11—C12—O1	−5.6 (4)	C33—C34—C35—C36	−0.6 (10)
C16—C11—C12—C13	−1.7 (4)	C32—C31—C36—C35	1.1 (6)
C1—C11—C12—C13	175.3 (3)	C8—C31—C36—C35	−175.9 (4)
O1—C12—C13—C14	−178.5 (3)	C34—C35—C36—C31	−0.2 (7)
C11—C12—C13—C14	0.6 (4)	C4—N2—C8—C31	−72.2 (4)
C12—C13—C14—C15	0.7 (5)	C1—N2—C8—C31	164.2 (3)
C13—C14—C15—C16	−0.7 (4)	C36—C31—C8—N2	−43.1 (5)
C12—C11—C16—C15	1.8 (4)	C32—C31—C8—N2	140.1 (4)
C1—C11—C16—C15	−175.3 (2)	C36'—C31'—C32'—C33'	−4 (3)
C14—C15—C16—C11	−0.5 (4)	C8'—C31'—C32'—C33'	175 (2)
N1—C7—C21—C22	57.9 (3)	C31'—C32'—C33'—C34'	5 (4)
N1—C7—C21—C26	−122.3 (3)	C32'—C33'—C34'—O3'	177 (2)
C26—C21—C22—C23	0.8 (4)	C32'—C33'—C34'—C35'	−4 (4)
C7—C21—C22—C23	−179.3 (2)	C37'—O3'—C34'—C33'	7 (3)
C21—C22—C23—C24	−0.2 (4)	C37'—O3'—C34'—C35'	−172.6 (18)
C22—C23—C24—O2	178.5 (3)	C33'—C34'—C35'—C36'	1 (3)
C22—C23—C24—C25	−0.9 (4)	O3'—C34'—C35'—C36'	−179.2 (13)
C23—C24—O2—C27	1.4 (10)	C34'—C35'—C36'—C31'	0 (2)
C25—C24—O2—C27	−179.2 (9)	C32'—C31'—C36'—C35'	2 (2)
C23—C24—O2—C27'	−26 (3)	C8'—C31'—C36'—C35'	−177.4 (13)
C25—C24—O2—C27'	153 (3)	C4—N2—C8'—C31'	−66.0 (14)
O2—C24—C25—C26	−178.2 (2)	C1—N2—C8'—C31'	157.2 (11)
C23—C24—C25—C26	1.3 (4)	C32'—C31'—C8'—N2	108.9 (18)
C24—C25—C26—C21	−0.6 (4)	C36'—C31'—C8'—N2	−72.1 (19)

### Hydrogen-bond geometry (Å, º)

*Cg*4 is the centroid of the C31–C36 ring (major occupancy component).

**Table:**

*D*—H···*A*	*D*—H	H···*A*	*D*···*A*	*D*—H···*A*
O1—H1···N1	0.92 (4)	2.01 (4)	2.740 (3)	135 (3)
C27—H27*B*···O3^i^	0.98	2.58	3.342 (10)	134
O4—H4···*Cg*4^ii^	0.84	2.96	3.723 (4)	151
O4′—H4′···O1^iii^	0.84	2.15	2.898 (6)	148
C27′—H27*D*···O4′^iv^	0.98	2.45	3.35 (6)	153
C35′—H35′···O2^iii^	0.95	2.55	3.254 (13)	131

Symmetry codes: (i) −*x*+1, *y*+3/2, −*z*+1/2; (ii) −*x*+1, *y*+1/2, −*z*+1/2; (iii) −*x*+1, *y*−1/2, −*z*+1/2; (iv) *x*, *y*+1, *z*.
